# Microfabricated Inserts for Magic Angle Coil Spinning (MACS) Wireless NMR Spectroscopy

**DOI:** 10.1371/journal.pone.0042848

**Published:** 2012-08-20

**Authors:** Vlad Badilita, Birgit Fassbender, Kai Kratt, Alan Wong, Christian Bonhomme, Dimitris Sakellariou, Jan G. Korvink, Ulrike Wallrabe

**Affiliations:** 1 Laboratory for Microactuators, Department of Microsystems Engineering (IMTEK), Albert Ludwigs University Freiburg, Freiburg, Germany; 2 UPMC Univ Paris 06 & CNRS, UMR 7574, Chimie de la Matière Condensée de Paris, Collège de France, Paris, France; 3 CEA Saclay/DSM/IRAMIS/SIS2M – CNRS UMR3299, Gif-sur-Yvette, France; 4 Laboratory for Simulation, Department of Microsystems Engineering (IMTEK), Albert Ludwigs University Freiburg, Freiburg, Germany; 5 Freiburg Institute of Advanced Studies (FRIAS), Freiburg, Germany; Imperial College London, United Kingdom

## Abstract

This article describes the development and testing of the first automatically microfabricated probes to be used in conjunction with the magic angle coil spinning (MACS) NMR technique. NMR spectroscopy is a versatile technique for a large range of applications, but its intrinsically low sensitivity poses significant difficulties in analyzing mass- and volume-limited samples. The combination of microfabrication technology and MACS addresses several well-known NMR issues in a concerted manner for the first time: (i) reproducible wafer-scale fabrication of the first-in-kind on-chip LC microresonator for inductive coupling of the NMR signal and reliable exploitation of MACS capabilities; (ii) improving the sensitivity and the spectral resolution by simultaneous spinning the detection microcoil together with the sample at the “magic angle” of 54.74° with respect to the direction of the magnetic field (magic angle spinning – MAS), accompanied by the wireless signal transmission between the microcoil and the primary circuit of the NMR spectrometer; (iii) given the high spinning rates (tens of kHz) involved in the MAS methodology, the microfabricated inserts exhibit a clear kinematic advantage over their previously demonstrated counterparts due to the inherent capability to produce small radius cylindrical geometries, thus tremendously reducing the mechanical stress and tearing forces on the sample. In order to demonstrate the versatility of the microfabrication technology, we have designed MACS probes for various Larmor frequencies (194, 500 and 700 MHz) testing several samples such as water, *Drosophila* pupae, adamantane solid and LiCl at different magic angle spinning speeds.

## Introduction

NMR spectroscopy is a cross-disciplinary powerful and versatile technique used in the investigation of the chemistry, structure and global architecture of chemically- and biologically-relevant samples. In spite of being a non-invasive, highly specific technique, NMR spectroscopy has an inherent disadvantage when it comes to mass- and volume-limited samples: it is a rather insensitive method since the magnetic resonance signal stems from the extremely small population difference between the upper and the lower energy states governed by the Boltzmann distribution.

### Improving the sensitivity of the NMR experiment

There is a long list of strategies to increase the intrinsic sensitivity of the NMR experiment, but probably the most significant research effort has been directed towards increasing the *B_0_* field, since the sensitivity increases as the 7/4^th^ power of the magnetic field strength [1]. In order to compensate for the extremely small population difference between the upper and the lower energy states, which typically amounts to less than 0.01% of the total number of molecules, another strategy employs polarization transfer from optically pumped nuclei (e.g., ^129^Xe) via spin polarization induced Overhauser enhancement, resulting in a 50 times increase in proton NMR signal strength [2].

Room for many improvements has also been found at the level of the RF coils, essentially exploiting the fact that the thermal noise in the coil itself rather than the sample represents the main limitation at the microscale. The coil quality factor can be increased and the thermal noise limited by using coil wire which is superconducting at elevated temperatures [3] or by cryogenic cooling [4] of the microcoils.

The sensitivity drawback is also significantly alleviated by downscaling the NMR detector to closely conform to the small target sample [5], [6]. A wide range of miniaturized designs have been reported with significant improvements in terms of sensitivity. The stripline probes for NMR [7], [8] combine high sensitivity with low susceptibility line broadening in a simple and scalable design. A planar microslot waveguide NMR probe [9] allows for the reduction of the required amount of sample by several orders of magnitude, at the same time providing excellent sensitivity and spectral resolution. In recent years, solenoidal microcoil detectors have become popular [10]–[12] exploiting the fact that the efficiency of such geometry is inversely proportional to the diameter of the solenoid [1], [10]. Our team has recently addressed the issue of detector miniaturization [13] by reporting a robust, batch fabrication process for 3D solenoidal microcoils with applications in microscale magnetic resonance imaging (MRI). The process relies on the unique capabilities of an automatic wirebonder in conjunction with techniques traditionally used for the development of micro-electro-mechanical systems (MEMS). We have also performed an in-depth characterization of the wirebonded microcoils for MRI purposes [14].

### Spectral resolution of the NMR experiment

Spectral resolution is another crucial aspect of NMR analysis. In any condensed phase, the nuclear spin interactions broaden the spectral lines, rendering them featureless and hard to interpret. A technique [15], [16] often used to increase the spectral resolution is magic angle spinning (MAS), which involves sample spinning up to 70 kHz about an axis tilted at the “magic angle” with respect to the external static magnetic field (B_0_). At the magic angle (θ_m_ = 54.74°), the time dependent nuclear dipole-dipole and first order quadrupolar interactions average to zero (3cos^2^θ_m_−1 = 0), while the other time dependent interactions (chemical shift anisotropy and J couplings) are averaged to non-zero isotropic values. Thus, in the MAS experiment, the normally broad lines become narrower, thereby increasing the resolution and sensitivity of the NMR experiment.

The MAS configuration uses a static coil to pick up the signal from a rapidly rotating sample. There are a couple of efforts to combine the versatility of MAS NMR spectroscopy with the increased sensitivity offered by the solenoidal microcoil detector geometry. Janssen et al. [17] take the device previously developed for static experiments [11] one step further inserting a rotor in the pick-up microcoil. A similar approach has been reported by Yamauchi [18] for solid state NMR for limited amounts of peptide crystals and protein samples.

However, the MAS approach brings back the sensitivity issue since the filling factor of the pick-up coil decreases. In order to circumvent this problem, a new NMR technique called “magic angle coil spinning” (MACS) involving fast spinning of both the sample and the miniaturized detector coil, together with inductive wireless coupling between the sensor picking up the NMR signal and the static coil has been previously demonstrated [19], [20], bringing one order of magnitude increase of the signal for small-sized samples when compared to standard NMR. This remarkable NMR setup requires direct winding of the coil around the sample capillary in order to optimize the filling factor. At the same time, the local magnetic field inhomogeneities introduced in the sample by the proximity of the wire are eliminated by spinning the microcoil at the magic angle, simultaneously with the sample.

Although the recently introduced [19] simultaneous spinning of microdetectors together with the sample (MACS) has marked a significant advancement in terms of sensitivity and resolution, for the moment its use remains restricted mainly because of the difficulties to fabricate a large number of such NMR probes with controllable and reproducible properties. The miniaturized detector coils have been previously manually wound, thus rendering the manufacturing tedious and affecting the reproducibility of their electrical characteristics [19]. At the same time the tuning capacitor has been soldered by hand as a discrete component, therefore the entire ensemble may also suffer from mechanical unbalance at high rotational speeds.

This paper presents the design and characterization of the first-in-kind on-chip microresonator for MACS NMR spectroscopy. We report herewith important advances in making robust and reproducible microfabrication technology part of the routine MACS methodology, thus circumventing the issues related to the mechanical stability and performance repeatability of the detector microcoils. The performance of the microfabricated MACS probes is assessed in terms of sensitivity, and the robustness is successfully proven at low (110 Hz) and high (up to 10 kHz) spinning speeds. The flexibility of the manufacturing technology is demonstrated by designing and testing the on-chip NMR probes for two different nuclei at three different Larmor resonant frequencies: ^7^Li at 194 MHz (11.7 T) and ^1^H at 500 MHz (11.7 T) and 700 MHz (16.4 T). We critically discuss the performance of the reported devices with respect to commercially available NMR probes as well as to various designs previously reported in the literature, clearly indicating the directions for further improvments.

## Methods

### Concept and microfabrication of the MACS NMR probe

**Figure 1:**
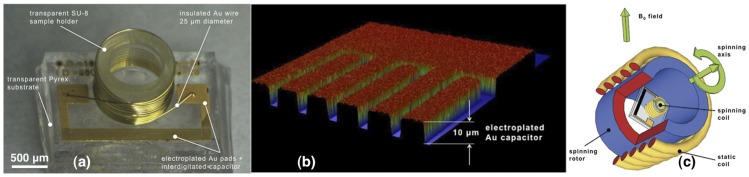
a) Microscope image of the microfabricated NMR insert. b) Profile of the electroplated on-chip capacitor. c) Schematic of the magic angle coil spinning (MACS) experimental set-up.

**The NMR probe reported here and shown in** **Figure 1a** **is an LC microresonator and consists of a solenoidal wirebonded micro**coil [21] integrated with an interdigitated capacitor patterned on-chip. The LC-circuit consisting of the capacitor and microcoil is designed to resonate at the Larmor frequency of interest, enabling wireless inductive coupling of the NMR signal, as well as spinning at the magic angle for high resolution NMR.

**The fabrication of the on-chip LC microresonators presented in this work is based on the previously presented process** [21], [22] for the fabrication of wirebonded solenoidal microcoils, taking advantage of standard microfabrication techniques, together with the unique capabilities of the automatic wirebonder. Briefly, a Pyrex wafer is sputtered with CrAu (50/500 nm), which is further electroplated in a photoresist mold to the desired thickness. As shown in Figure 1b, the total thickness of the electroplated gold is 10 µm in order to compensate for the skin depth of gold at 500 MHz. The seed layer is further etched away and at this point the interdigitated capacitors of the LC micro-resonator, as well as the contact pads for subsequent wirebonding of the microcoils, are completely defined. In order to minimize the footprint of the entire chip, the physical dimension of the interdigitated capacitor must be kept as small as possible. Therefore, the next step in the process flow is to embed the capacitor fingers in a block of SU-8 epoxy defined by UV lithography, thus increasing the capacitance of the interdigitated capacitor by a factor equal to the SU-8 relative permittivity (ε_r_ = 4). To this end, SU-8 3050 UV-sensitive epoxy is spun on the wafer to a thickness of 50 µm and patterned on top of the capacitor fingers. Next, 650 µm high hollow cylinders are defined by UV lithography of high viscosity epoxy SU-8 2150 to serve both as sample containers and as mechanical support for microcoil wirebonding, according to the previously reported method [13].

Microcoil winding is the final step in the process flow. The coil parameters, i.e., the number and the pitch of windings, are chosen so that the microcoil, together with the on-chip interdigitated capacitor, resonate at the desired frequency. In this work we consider on-chip NMR microresonators designed and tested for three different Larmor frequencies: 500 MHz and 700 MHz corresponding respectively to proton ^1^H resonance at 11.7 T and 16.4 T, and 194 MHz corresponding to ^7^Li at 11.7 T. The device shown in the optical microscope image in Figure 1a is a typical NMR microresonator for 500 MHz with 13 windings and 5 interdigitated fingers.

### Electrical characterization of the microfabricated probe and MACS experimental setup

For the microcoil design we use the Wheeler formula [23] since we have previously demonstrated an excellent agreement between the values predicted with this formula and the actual values of the microcoils fabricated using the present wirebonding technology [21]:
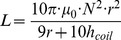
where μ_0_ = 4π×10^−7^ H/m is the magnetic permeability of free space, *N* is the number of windings, *r* is the coil radius and *h_coil_* is the coil height (the microcoil in Figure 1a has a radius of *r* = 500 µm, *h_coil_* = 650 µm and *N* = 13 windings yielding *L* = 190 nH). For this value, the on-chip interdigitated capacitor is calibrated to 0.53 pF.

A schematic of the MACS experiment is given in Figure 1c. The NMR on-chip coil fabricated as detailed above is glued on a cylindrical Shapal-M ceramic insert, machined to place the coil at the center of the rotor. This insert fits tightly inside a commercial rotor so that the coil and the sample are placed along the rotation axis thus having a constant electromagnetic coupling during spinning. The rotation axis is tilted at the magic angle (θ_m_ = 54.74°) with respect to the B_0_ field of the NMR spectrometer. After placing the rotor containing the microresonator inside the MAS probe, the wobbling curve may indicate one or two resonances depending on the coupling regime between the microcoil and the static coil [24]. The tuning and matching elements of the spectrometer are used to perform the fine-tuning to the exact Larmor resonant frequency and to match the ensemble to 50 Ω. The experimental setup is based upon the wireless inductive coupling between the static coil of the commercial MAS probe, which is normally used for spin manipulation and signal reception, and our on-chip microcoil tuned at the resonant frequency. As explained above, the microcoil is wound around the SU-8 sample container. Therefore it rotates in concert with the sample.

**Figure 2:**
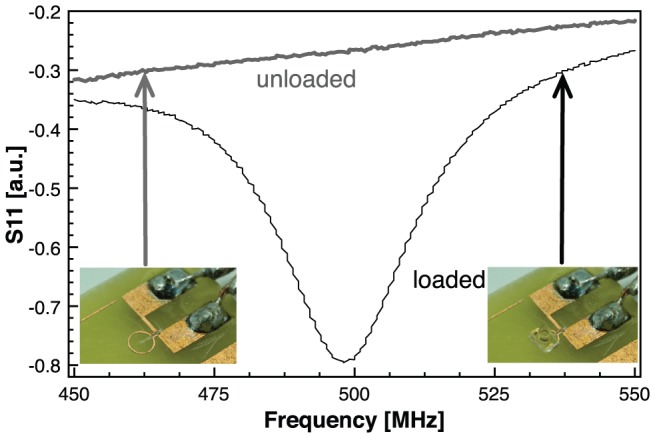
Successful tuning of the microfabricated NMR insert at 500 MHz.

The tuning of the NMR probe is tested immediately upon fabrication in a very simple and precise set-up. The return loss spectrum (S_11_ parameter) of a PCB loop coil is measured both unloaded and loaded with the wirebonded microresonator. For a successfully tuned NMR probe, the return loss spectrum of the loaded PCB loop coil should exhibit a strong absorption at the desired frequency as shown in Figure 2 for 500 MHz. The typical deviation from the target resonant frequency of the fabricated microresonators is below 3%.

### NMR spectroscopy measurements

**Figure 3:**
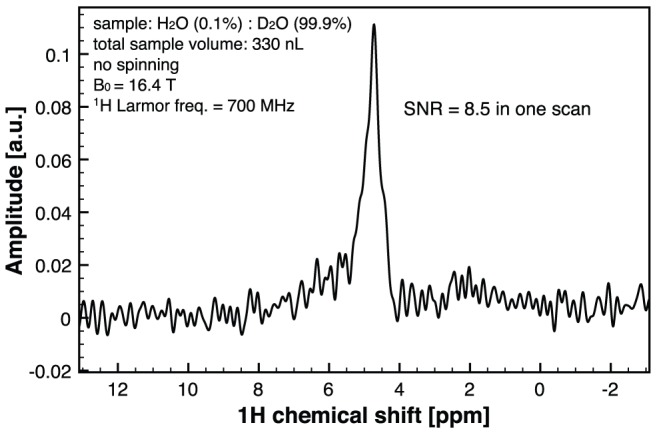
Sensitivity of the microfabricated NMR insert. ^1^H spectrum at 700 MHz of a mixture of 99.9% D_2_O and 0.1% H_2_O.

In order to test the sensitivity of the probe via inductive coupling, the NMR experiment shown in Figure 3 has been performed on a narrow bore 16.4 T Bruker Avance III spectrometer, using a 3.2 mm MAS probe. Deuterated water (99.9% D_2_O and 0.1% H_2_O) was used to test the detection limit. The Larmor frequency of the protons at 16.4 T is 700 MHz, therefore the LC microresonator was designed to resonate at this frequency.

**Figure 4:**
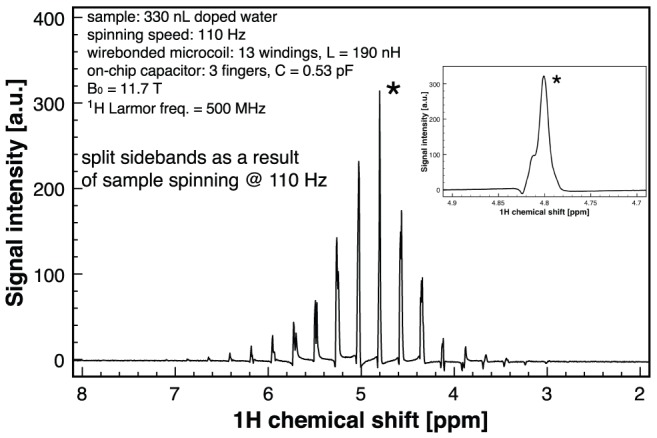
500 MHz ^1^H NMR spectrum of 330 nl water spun at 110 Hz. The isotropic central peak is split in sidebands spaced at 110 Hz, i.e., 0.22 ppm at 500 MHz. The inset shows the central water peak which has a non-Gaussian shape due to the field inhomogeneities (see *Discussion* section).

**Figure 5:**
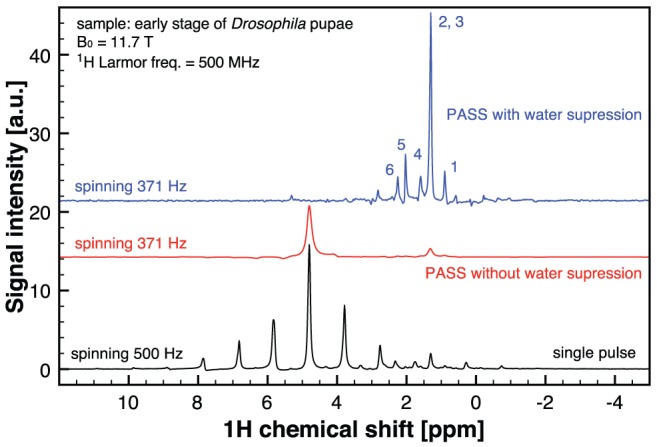
^1^H NMR spectra of Drosophila pupae. The spectra was acquired with a total of three pupae in a 400 µm-microcoil coupled with a standard 7-mm MAS probe, spinning at 500 Hz for the single-pulse experiment, and at 371 Hz for the PASS experiment. The total experimental time for the PASS experiment is about 1 hour. Peak assignment (1) Lipid –CH_3_ (2) fatty acid –(CH_2_)_n_ (3) lactate CH_3_ (4) Lipid –CH_2_-CH_2_-CO– (5) Lipid–CH = CH-CH_2_-CH_2_– (6) Lipid –CH_2_-CH_2_-CO–.

The slow spinning speed experiments (Figure 4), the high speed spinning spectrum of adamantane (Figure 5) as well as the ^7^Li spectrum (electronic supplementary information) have been performed in a wide bore 11.7 T Bruker Avance II spectrometer using a standard 7 mm HX CP-MAS probe. The ^1^H Larmor frequency at 11.7 T is 500 MHz, while for ^7^Li is 194 MHz. Therefore the LC microresonators used in these experiments were designed to resonate at these frequencies, respectively.

## Results

### Sensitivity of the MACS NMR probe is uncompromised in the inductive coupling configuration

In order to assess the sensitivity of the NMR probe in the MACS NMR configuration, the sample container has been filled with a mixture containing 99.9% heavy water – D_2_O and 0.1% H_2_O. The only NMR signal will come from the 0.1% water in the sample container. Taking into account the dimensions of the SU-8 sample container specified above, the entire sample volume is 330 nl, therefore only 330 pl of H_2_O is responsible for the NMR signal. Figure 3 shows the static (without sample spinning) NMR spectrum taken in a 16.4 T magnetic field, which corresponds to a Larmor frequency of the proton of 700 MHz. The signal to noise ratio (SNR) of a single scan experiment is 8.5, proving an excellent sensitivity of the on-chip NMR probe in this configuration.

Utz et al. [25] have previously demonstrated that the microcoil sensitivity is essentially uncompromised in the inductive coupling configuration. Starting from the general principle that the signal to noise ratio (*SNR*) intrinsic to the detector coil verifies:
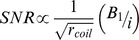
where *r_coil_* is the ohmic resistance of the detector coil and *B_1_* is the field produced by the current *i* in the detector coil, the signals observed respectively when the microcoil is tethered – *(SNR)_tethered_*, and when the microcoil is inductively coupled (wireless) – *(SNR)_wireless_*, can be expressed respectively by:
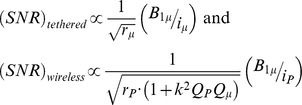
where index *μ* denotes the microcoil (either tethered or in inductive coupling configuration), and index *P* denotes the probe coil, *Q* is the quality factor and *k* is the coupling constant between the microcoil and the probe coil given by:
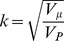

*V_μ_* and *V_P_* are the volumes of the microcoil and the probe coil, respectively. In order to estimate the sensitivity penalty to be paid in the case of inductive coupling configuration, one has to evaluate the following ratio [25]:
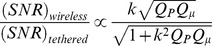
In the particular case of our experimental setup, the volume of the static coil is 74.8 µl while the volume of the microcoil is 0.51 µl (the microcoil diameter is 1 mm and the microcoil height is 650 µm). The measured typical quality factors of the wirebonded solenoidal microcoils have exceeded the value of 40 [21] and the usual value for the quality factor of the probe coil is *Q_P_≈200*. Therefore the coupling constant between the two coils is *k = 0.082* and the above-mentioned ratio is evaluated:
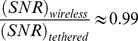
which demonstrates that the wireless inductive coupling configuration does not affect the sensitivity of the NMR experiment if compared with the direct coupling configuration.

### Limit of detection of the MACS NMR probe

The SNR value given in Figure 3 is affected by many factors related to the size and geometry of the detection coil, the volume and concentration of the sample, as well as the scan parameters, including the number of acquisitions and total acquisition time (*t_acq_*). In order to give a valid characterization of a certain probe one has to specify standard conditions for data collection and processing since for a particular sample and experimental setup, the NMR spectrum depends on acquisition and processing parameters.

For the case of mass- and volume-limited samples it is often required that a number of acquisitions must be accumulated or the concentration must be increased to yield a meaningful result. Therefore, we propose to use the parameters introduced by Lacey et al. [26] for time-normalized mass sensitivity (*S_m_*) and normalized limit of detection (*nLOD_m_*), respectively:
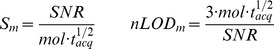
where *mol* refers to the mole amount of the sample which resides within the active volume of the microcoil and *t_acq_^1/2^* accounts for the total time of the experiment. These values are helpful to the NMR spectroscopist in finding the best trade-off between potential gains in SNR and a reduced data acquisition time and/or sample quantity. The normalized limit of detection is also defined in terms of sample mass and specifies the minimum mass yielding an SNR value of 3. Normalized limits of detection are a measure of probe sensitivity but also an approximate indicator for the NMR spectroscopist of the sample mass or concentration needed to achieve a certain SNR value for a specific spectral signal.

By performing all the calculations with the results presented in Figure 3 at 700 MHz, the mass sensitivity of our on-chip NMR probe is *S_m_* = 230 µmol^−1^·Hz^1/2^. In order to perform a meaningful comparison with experimental performances of other NMR probes, we have chosen the 5 mm Varian probe and the reduced diameter saddle coil probe from Nalorac investigated by Lacey et al. in Ref. [26]. We converted the values from Ref. [26] into number of spins since these tests have been performed with respect to the signal from the anomeric proton of sucrose while in the present work water has been used as sample. Also, the results presented here are obtained at 700 MHz as opposed to 600 MHz in Ref. [26], therefore normalization is required for appropriate comparison. The signal amplitude varies as a function of 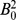 and due to the skin effect the microcoil resistance varies as the square root of the frequency. Therefore the SNR is proportional to 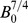. Taking all these facts into account, the limit of detection for our on-chip NMR probe is *nLOD_m_* = 2·10^16^ spin·Hz^−1/2^ which is the same order of magnitude as the two commercial probes evaluated in Ref. [26]. These results show that our on-chip probes are comparable with commercial standards, although miniaturization is expected to further increase the sensitivity.

### MACS NMR probe at low spinning speeds

The magic angle coil spinning technique (MACS) increases the filling factor of the detector coil by winding it directly around the sample holder, at the same time retaining the advantage to eliminate the bulk magnetic susceptibility of the wire of the microcoil by spinning at the magic angle. It is also well known that fast spinning brings along better spectral resolution. However, spinning the sample and the entire detector coil (in this case wire and metal pads) in a very strong magnetic field (the B_0_ field) generates eddy currents, which may thermally affect both the sample and the coil. In a previous detailed study [27] of the effects of spinning in the MACS configuration it has been demonstrated that the temperature increase shows a dependence upon the third power of the radius of the wire used in microcoil fabrication and upon the second power of the spinning speed. It is therefore beneficial to use low spinning speeds in order to avoid heating especially for applications involving biopsies which can be damaged at high temperatures or NMR spectroscopy of small molecules which exhibit temperature dependent shifts such as water and methanol. However, as described later in the text, the use of microfabrication technologies along with thin coil wire significantly alleviates the heating issue for this NMR probe.

In order to assess the NMR performance of the setup at low rotation speed, a probe filled with 330 nl CuSO_4_-doped water sample has been spun at 110 Hz. CuSO_4_-doped water (50 mM concentration) has been used in order to reduce the T_1_ relaxation time and increase the signal. Figure 4 presents a MACS spectrum of the doped water sample. One can see the split sidebands as a result of sample spinning. The separation of the sideband peaks is given by the spinning frequency, in this case 110 Hz, i.e. 0.22 ppm at 500 MHz. Higher spinning speeds would increase the sidebands spacing rendering easier to interpret a spectrum which is potentially more complicated, e.g., of a biopsy sample with various different yet close metabolite signals. However, well-established synchronization techniques for total suppression or separation of the spinning sidebands [28] can be employed in order to separate the information contained in the central, isotropic peak of the spectrum [28], [29] thus making slow speed NMR a useful tool for samples where high spinning speeds are prohibited.

It has recently been demonstrated that the use of hand-wound MACS shows great potential for metabolic profiling in tissue biopsies [30]. To evaluate the wirebonded MACS inserts for metabolomic NMR spectroscopy, we have acquired ^1^H NMR spectra of early stage Drosophila pupae, see Figure 5. To preserve the tissue integrity from the centrifugal forces generated from the sample spinning, we performed slow spinning speed experiments. A similar approach has been demonstrated with the hand-wound MACS [31]. At 500 Hz spinning speed, the single-pulse spectrum displays a large spinning sideband manifolds, which prevents revealing any metabolite signals of the pupae. However, when the sideband manifolds are suppressed by PASS, the spectrum clearly reveals two isotropic signals without sidebands at 4.7 ppm of water and at 1.3 ppm of lipid. Water suppression was also applied along with the PASS experiment in order to reveal the typical NMR signature of the lipid. It is noteworthy that the signal enhancement (per unit mass) gained by the microcoil is about 5. This is based on the reciprocity principle [1]: *B_1_* (wirebonded MACS coil)/B_1_ (MAS coil). As a result, the experimental time (ca. 1 hr) for the spectra shown in the figure is reduced by a factor of about 25. In other words, without the use of the microcoil, over 25 hours would be needed to acquire spectra with the same signal-to-noise level. In such lengthy experiment, the metabolic profiles in the pupae could change and could lead to inaccurate analysis of the metabolic activity.

### MACS NMR probe at high spinning speeds

**Figure 6:**
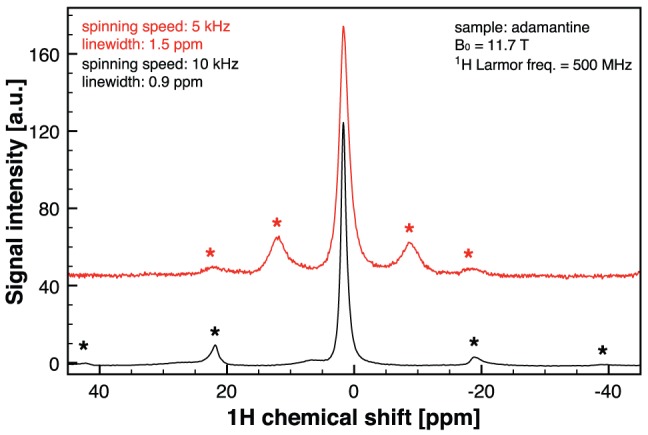
Linewidth narrowing of solid-state adamantane spectrum for higher rotational speeds. NMR spectra at 500 MHz for two different spinning speeds: 0.9 ppm at 10 kHz vs. 1.5 ppm at 5 kHz.

In order to characterize the behavior of the NMR probes for potential applications using high spinning rates, as for instance in solid state spectroscopy, we have acquired spectra of adamantane, a reference in NMR spectroscopy. To this purpose the spinning rate of the probes together with the sample has been significantly increased up to 5 kHz and 10 kHz. It is a remarkable result that the microcoils mechanically withstand both the large centrifugal forces arising at these spinning speeds and the heat dissipated due to the fast movement of conductive parts in a strong magnetic field. The adamantane spectra for these two spinning speeds are presented in Figure 6 where the double sideband separation at 10 kHz versus 5 kHz is visible, as well as a significant narrowing of the linewidth: 0.9 ppm at 10 kHz versus 1.5 ppm at 5 kHz.

The freedom in defining a variable number of both capacitor fingers and coil windings translates into the flexibility to design an NMR probe for various nuclei targeting different applications. Since solid state NMR has been very important in characterizing Li-based materials [32], [33], which became very popular once Sony had launched in 1991 the first rechargeable Li-ion battery, and in order to demonstrate the flexibility of our design, we present in the Electronic Supplementary Information (ESI) a spectrum for a spin 3/2 nucleus – Lithium (Figure S1). These results show that our NMR probe can be designed for a large range of resonant frequencies – the Larmor frequency for ^7^Li in an 11.7 T *B_0_* field is 194 MHz.

## Discussion

In this paper we have reported the design, manufacturing and testing of the first microfabricated insert for MACS NMR spectroscopy. The conclusions of this study will contribute to benefit future improved designs of microfabricated MACS inserts. We have identified several directions of future improvements.

### Susceptibility and sensitivity issues

There is a relatively large susceptibility mismatch among the different constituent materials of the NMR probe, mainly because of the Au in the pads, capacitor and coil wire. Both the sidebands in Figure 4 and the non-perfect Gaussian water signal and the absence of *J*-splitting in the sucrose spectrum (see Figure S2 – ESI) indicate *B_0_* field inhomogeneities introduced by the NMR probe. The magnetic susceptibility of gold is χ_Au_ = −34×10^−6^ as opposed to the other materials of the order −10^−5^ for the SU-8 container and any water-based sample. Several solutions for further improvement of the on-chip NMR probe are underway and involve replacing of the thick electroplated gold with copper for all the metallic structures on-chip and, in a next step, replacing the gold wire with copper wire. However, this solution requires a more intense technological effort because of the oxidation problems associated with copper during the wirebonding process. An additional potential solution to alleviate susceptibility mismatch issues is readily offered by the planar technology used to fabricate the on-chip metallic components, and involves a design of the tuning capacitor with a radial symmetry around the coil, i.e. around the MACS rotational axis.

Regarding the sensitivity of our probe calculated from the measurement presented in Figure 3, several limiting factors have been identified. The wirebonded solenoidal coils employed here have a rather small aspect ratio (height versus outer diameter) of 0.65 compared to relatively long coils reported in [11] with an aspect ratio of 2.5. This translates into a more homogenous *B_1_* field of the latter device, as well as increased sensitivity due to the smaller diameter. Moreover, the technology for wirebonded microcoils involves 25 µm Au wire as opposed to 50 µm Cu wire used in [11]. Nevertheless, as previously reported in [22], the fabrication technology employed here allows in principle to increase the aspect ratio of the detector coil structure either by SU-8 photolithography or, if much larger aspect ratios are required, by X-Ray lithography of PMMA instead of SU-8 [22]. Replacement of the gold wire with copper is also underway with benefits not only in improving the overall susceptibility matching issues of the entire structure as outlined above, but also in decreasing the resistance of the probe.

### Fundamental advantages of microfabricated MACS inserts

Several previously reported microdetector designs specifically target the high sensitivity and spectral resolution issues of the NMR experiments, therefore superior LOD values for very specific designs have been demonstrated. Kentgens et al. [11], [17] focus on the probe design to minimize losses from the coil leads and the tuning capacitors by mechanically integrating the solenoidal coil into a custom made capacitor. The achieved sensitivity is 10^14^ spins·Hz^−1/2^ for a signal-to-noise ratio of 1 in a single scan. The stripline probes [7], [8], [34] for NMR combine high sensitivity with low susceptibility broadening in a simple and scalable design reporting one order of magnitude further improvement in the sensitivity. An innovative design based on a planar microslot waveguide [9] significantly decreases the amount of sample to be used while preserving an excellent sensitivity and spectral resolution. Further designs include hand-wound solenoidal microcoils [10], [19] with well-known characteristics.

In order for an NMR microdetector to be used in a magic angle coil spinning experiments or equivalent, i.e., involving simultaneous spinning of both the sample and the microdetector, it has to meet several requirements:

- the NMR signal needs to be coupled wirelessly to the primary circuit of the spectrometer, therefore the microdetector design has to include an efficient inductive coupling feature;
- the entire design must be compact in order to be compatible with the typically small spaces inside the NMR spectrometers;
- given the fact that MAS experiments typically involve high spinning rates in the tens of kHz range, the stress and tearing forces acting on the samples become very important, therefore they need to be minimized via the microdetector final geometry;
- the NMR microdetector should have a good quality factor in order to offer a high sensitivity.

While the above-mentioned requirements are rather straightforward, let us focus on the kinematic aspect. One issue associated with the high speed spinning NMR is represented by the inherent large centrifugal forces acting on the detector coil and on the sample itself. For this reason, high speed MAS has been especially prohibitive for soft tissues (biopsies) and in-vivo metabolomics due to the centrifugal forces acting on the sample that can rupture the cells and damage the biological tissue. However, the miniaturization of the NMR probe is expected to significantly alleviate this problem. The stress (*σ*) in a rotating sample contained in an axial tube experiences its highest radial tensile stresses in the centre of the sample, on the axis, and its highest radial and tangential (hoop) compressive stresses at the container wall (which constrains the centrifugal forces from expanding the sample indefinitely). In both cases, the stresses are proportional to *f^2^R^2^*, where *f* is the spinning frequency and *R* is the radius of the sample. For a given maximum tensile or compressive stress level, a reduction in radius *R* allows a proportional increase in rotational frequency *f*.

Of all the designs, only the hand-wound microcoils have been reported in [19] to be used in magic angle coil spinning experiments, with the inductive coupling being achieved via a discrete capacitor manually soldered at the terminals of the coil. This detail has negative consequences on the mass balance and mechanical stability of the entire insert, which become important at high spinning rates. The stripline [7], [8], [34] and microslot waveguide probes [9] could in principle achieve the inductive coupling function only via a rather large additional coupling electronics module rendering the entire device bulky, therefore prone to failure at high spinning rates. Thus, even if the sensitivity is superior at this point, neither the stripline nor the microslot waveguide are compatible design for the MACS experimental setup.

The other typical concern in the MACS NMR configuration is the heating due to eddy currents. As previously demonstrated [27], there is a strong dependency between the temperature increase in the sample and the wire diameter, thinner wires leading to less heat dissipation. Aguiar et al. [27] have shown that for Cu wire with 25 µm diameter, the effective heating of a sample spinning at 6 kHz is rather small, around 2.4 degrees. Therefore, the miniaturization of the MACS probe extends the range of spinning speeds where sample heating is not an issue for the spectral accuracy, making this technique potentially useful for solid state applications. Since handling wires with small diameters is inherently difficult, the robust microfabrication technique presented here brings along a significant reduction in wire size for manufacturing MACS inserts. Along the same line of minimizing the unwanted effects of eddy currents generated because of the rapid movement in a strong magnetic field, a typical problem in the MAS NMR of conductive solid state samples is the impossibility to achieve high spinning speeds because the conductive sample itself acts as a perfect brake. Minimizing the amount of conductive material is expected to decrease the brake effect, i.e., is expected to minimize the heat dissipated in the volume of the sample via the eddy currents. Moreover, ultra-small samples have an additional advantage: the large surface-to-volume ratio inherently enhances the cooling rate of the system. This will allow MACS measurements at high spinning speeds, which are essential for line narrowing of solid samples.

Microfabrication technologies open the perspective for fast, cheap, reproducible fabrication of robust NMR microdetectors for high resolution, high sensitivity spectroscopy of mass- and volume-limited samples. Miniaturization is a viable solution for stable spinning as well as RF field amplification and sensitivity. At the same time, the smaller probe radius is reducing the centrifugal effects on live samples. This paper shows that the MEMS processing adapted to MACS has the potential to further improve performance and push the limits of detection achievable by this technique.

Miniaturization through microfabrication of the MACS inserts is expected to provide much more beyond fast, robust and reproducible means of large-scale manufacturing. At this time, this proves to be the technology of choice to fabricate small radius miniaturized NMR detectors, naturally lending itself for use in the magic angle coil spinning arrangement.

## Supporting Information

Figure S1 ^7^Li dynamic spectrum at 500 Hz spinning speed at 11.7 T. The MACS probes can be designed for a large range of Larmor frequencies. (for ^7^Li at 11.7 T – 194 MHz).
(EPS)

Figure S2 1D ^1^H water-suppressed NMR spectrum of sucrose in D_2_O. The spectrum has been acquired with the wirebonded MACS insert in 16 scans.
(EPS)
